# Voltametric Analysis of Ergosterol Isolated from Wild-Growing and Cultivated Edible Mushrooms from Serbia and Korea

**DOI:** 10.3390/molecules30092010

**Published:** 2025-04-30

**Authors:** Svetlana Đogo Mračević, Jelena Mutić, Vesna Stanković, Slavica Ražić

**Affiliations:** 1 Faculty of Pharmacy, University of Belgrade, Vojvode Stepe 450, 11221 Belgrade, Serbia; slavica.razic@pharmacy.bg.ac.rs; 2 Faculty of Chemistry, University of Belgrade, Studentski Trg 12-16, 11158 Belgrade, Serbia; jmutic@chem.bg.ac.rs; 3 Institute of Chemistry, Technology and Metallurgy, University of Belgrade, Njegoševa 12, 11000 Belgrade, Serbia; vesna.stankovic@ihtm.bg.ac.rs

**Keywords:** mushrooms, phytosterols, square-wave voltammetry (SWV), microelements, inductively coupled plasma atomic emission spectrometry (ICP-OES), principal component analysis (PCA)

## Abstract

Thanks to several components with health-promoting properties, mushrooms are recognized as a practical functional food and a valuable source of nutrients for the food industry. Ergosterol, the major sterol in edible mushrooms and a precursor of vitamin D2 with proven pharmacological activity and nutritional value, has become a very important topic in chemical and medical research. The main objectives of this study were to determine the ergosterol content in different species of Serbian wild mushrooms and in commercial mushrooms from Korean and Serbian grocery stores using square-wave voltammetry, to compare the concentrations in different parts of white button mushrooms, and to determine a possible relationship between Zn, Cu and Fe and ergosterol contents. The ergosterol contents varied between 0.01 and 7.04 mg/g (dry mass) of the mushrooms and were generally higher in cultivated mushrooms than in wild mushrooms. In addition, the ergosterol concentration was higher in the stems than in the caps of the mushrooms examined. Iron, Zn and Cu contents varied between the mushroom species at 8.5–479.9, 13.1–149.7 and 1.62–93.03 mg/kg, respectively, and principal component analysis (PCA) extracted two factors explaining 79.14% of the total variance, suggesting a direct relationship between iron and ergosterol content. This is the first comprehensive study to analyze and evaluate ergosterol concentrations in edible mushrooms from Korea and Serbia.

## 1. Introduction

The valorization of bio-waste and its use as a primary source to obtain value-added products are strategies that are equally applied to edible mushrooms, regardless of whether they grow wild or are cultivated under controlled conditions. The growing interest in mushrooms is based on general knowledge and the increase in new scientific evidence of their bioactivity and nutritional value.

**Figure 1:**
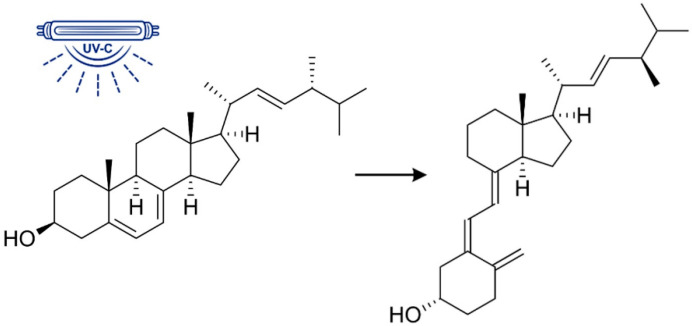
Conversion of ergosterol into ergocalciferol (vitamin D2).

In general, mushrooms are rich in bioactive compounds, a significant proportion of which belong to the phytosterols, including ergosterol (ergosta-5,7,22-trien-3β-ol), a major product of sterol biosynthesis that plays an essential role in the aerobic growth of fungi (Figure 1).

There are numerous scientific reports on its bioactivity. It is important to distinguish between the health-promoting effects of the mushroom extract, which is a very complex mixture of different compounds with a high variability depending on the extraction technique [1,2], and the bioactivity of the individual ergosterol.

Ergosterol is recognized as a bioactive molecule with proven anti-inflammatory and antimicrobial activity [3,4]. In addition to antimicrobial and antioxidant properties, antidiabetic and antineurodegenerative properties are also observed in edible mushrooms [5]. It has also been reported that ergosterol can repair oxidative damage in cells and reduce reactive oxygen species [6]. In the same study, ergosterol isolated from metabolites of fermented *Monascus anka* in media with *Dendrobium nobile Lindl* showed a good inhibitory effect on lipid peroxidation in a test of antioxidant activity in vitro. Modulation of protein expression and metabolic profiling by ultra-high-performance liquid chromatography–electrospray ionization mass spectrometry (UHPLC-ESI-QMS) confirmed that ergosterol extracted from *Leucocalocybe mongolica* exhibited anticancer activity against breast cancer cells [7].

Another important aspect is the ultraviolet radiation-assisted conversion of ergosterol to ergocalciferol, i.e., vitamin D2, by photochemical bond breaking of a steroid (Figure 1) [8,9,10]. When the mushroom powder was directly irradiated with UV-C radiation (254 nm), a 16% decrease in ergosterol and thus an increase in vitamin D2 was observed. A similar trend was observed when it was irradiated with UV-B (310 nm), while no conversion of ergosterol to vitamin D2 was observed when it was irradiated with UV-A (365 nm) [10].

Vitamin D is used as a complementary substance to fortify foods or to develop new functional foods with hypocholesterolemic properties. Its role in the human body is of paramount importance for calcium and phosphorus metabolism, and its deficiency leads to many serious diseases, such as osteoporosis, autoimmune diseases, diabetes, cancer and other serious health problems [9,10]. Mushrooms are therefore a good source of ergosterol with an additional value that can be used for the production of ergocalciferol.

Mushrooms, both wild and cultivated, are nutritious foods with excellent pharmacological and nutritional properties: low in calories and rich in fiber, vitamins, minerals, some β-glucans, antioxidants and flavorings [11].

Mushrooms contain high levels of ergosterol and have been used as food and medicine for centuries. Although more than 2000 edible mushroom species are known, button mushrooms (*Agaricus bisporus*) make up around 90% of the mushrooms currently cultivated and consumed in Europe. In Asia, on the other hand, shiitake, enoki and oyster mushrooms are very popular.

The ergosterol content in mushrooms was investigated by Mattila et al. [12], who determined values of 6.54 mg/g dry matter (DM) and 6.02 mg/g DM for white and brown mushrooms, respectively. The total amount of ergosterol in the different mushroom species varied. The highest ergosterol content was found in button mushrooms (7.80 ± 0.35 mg/g DM) and the lowest in enoki mushrooms (0.68 ± 0.14 mg/g DM) [13]. In addition, the authors found that the distribution of ergosterol content in different parts of the shiitake mushroom was different and accumulated more in the gills than in the stems.

The ergosterol content of wild and cultivated mushroom samples was determined using square-wave voltammetry (SWV) [14]. A fast, reliable and cost-effective method for Erg detection was developed based on the use of an unmodified boron-doped diamond electrode. This method is a fast and inexpensive, but satisfactory, sensitive and selective method for the quantification of ergosterol.

Moreover, mushrooms are considered to be mineral-rich products; K and P are the two most abundant elements in mushroom fruiting bodies, followed by Ca, Mg, Na, Fe, Zn and Cu [15]. In recent years, information has emerged on the contents of minerals in wild and commercialized mushrooms [11,16,17].

In addition, fungi can accumulate, store and tolerate relatively high concentrations of potentially toxic elements from the substrates on which they grow and have evolved a number of mechanisms to control and respond to high concentrations of heavy metals [18]. One of these responses is the formation of reactive oxygen species, which leads to damage of proteins and enzymes [18,19]. Considering the role of Fe and Zn as cofactors of metalloenzymes in the biosynthesis of ergosterol [20] and possible negative effects of high Cu concentrations on the ergosterol biosynthetic pathway on the one hand and antropogenic pollution sources on the other, this study was extended to the determination of these three elements by inductively coupled plasma optical emission spectrometry (ICP OES).

The main objectives of this study were as follows: (1) the determination of the ergosterol content in different varieties of Serbian wild mushrooms and in commercial mushrooms from Korean and Serbian grocery stores using the voltametric method; (2) the determination of the ergosterol content in different parts of Serbian button mushrooms; and (3) the determination of the concentrations of Zn, Cu and Fe to find a correlation between microelements and ergosterol content. To the best of our knowledge, this is the first comprehensive study to analyze, evaluate and compare ergosterol contents in edible mushrooms from two distant countries, Korea and Serbia.

## 2. Results and Discussion

The determination of the ergosterol content in different types of commercial edible mushrooms from Korean and Serbian grocery stores and in Serbian wild mushrooms was carried out using the voltametric method. The results are shown in Table 1. The analyzed mushroom samples showed significant variation in ergosterol content (0.01–7.04 mg/g). Wild mushrooms had the lowest ergosterol content, with a mean value of 0.9 mg/g. Significant variability in the sterol content in edible mushrooms (cultivated and wild mushrooms) has also been found previously [21].

In general, the wild mushrooms, with the exception of *B. fechtneri* (7.04 mg/g) and *L. crocipodium* (1.06 mg/g), contained less than 1 mg/g ergosterol. In the samples of *B. impolitus* and *P. rhodoxanthus*, the ergosterol content was below the detection limit. As there is a lack of data for the same species of edible wild mushrooms, we could only compare the results with other species: *Ganoderma lucidum* contained 1.20 mg/g free ergosterol [22], *Cordyceps sinensis* 1.60 mg/g [23], *Agrocybe aegerita* 2.45 mg/g and *Termitomyces albuminosus* 2.62 mg/g [24]. *Chantarellus cibarius* had 2.06 mg/g, *Chantarellus tubaeformis* 3.82 mg/g, *Lactarius trivialis* 3.03 mg/g and *Boletus edulis* 5.18 mg/g [12,25].

In the cultivated edible mushrooms, the ergosterol concentration varied between 0.54 and 6.96 mg/g for Korean enoki and button mushrooms, respectively. The average ergosterol concentration of 2.90 mg/g generally corresponded to the mean value for edible mushrooms reported worldwide (Table 2). The ergosterol content in shitake mushrooms (5.9 mg/g) was similar to previously reported values [21,26,27] but higher than the mean value reported by Jung et al. [28]. The mean ergosterol content in Jew’s ear mushrooms reported by Xu et al. [27] was two times higher than that obtained in the present study. The ergosterol content in oyster mushrooms was similar to that reported by Nzekoue et al. [29] and about two times lower than that reported by Saini et al. [21] and Slawinska et al. [30] for this type of mushroom.

There was no significant difference in ergosterol content between Serbian (1.14–4.67 mg/kg) and Korean (1.90–6.96 mg/g) white button mushrooms. The ergosterol content seems to be more dependent on the producer or type of substrate than on the country of origin. In the studies shown in Table 2, the ergosterol levels in this type of mushroom ranged from 0.11 to 9.89 mg/g, depending on the preservation method. Guan et al. [32] found that the ergosterol content in brown mushrooms is higher than in white mushrooms. The ergosterol concentration and stability is often more influenced by the post-harvest treatment and storage of mushrooms than by the conditions in the growing area [32,34]. Ergosterol biosynthesis has been observed to continue for up to 14 days during post-harvest storage at 4 °C under dark conditions at 95–100% humidity [32]. The food industry uses UV technologies for the surface sterilization of products [32]. For example, UV-C can damage the DNA of living organisms, which leads to the denaturation of proteins and the suppression of microbial growth and reproduction [35]. At the same time, UV irradiation ensures the conversion of ergosterol into vitamin D2 [13,27,34,36]. Different wavelengths of UV irradiation have different effects on this conversion process. In general, UV-C and UV-A are more efficient than UV-B [36]. UV-C showed the greatest D2 increase, but this varied among different fungal species [36]. Numerous studies have reported that UV treatment decreases ergosterol concentration and increases vitamin D2 concentration in fresh mushrooms, with a direct response to UV-C dose and irradiation time [28,33,37]. In addition, the conversion of ergosterol to vitamin D is optimal at a high moisture content (70–80%), which is indicative of fresh mushrooms [13]. Due to their high water content (87–95%), mushrooms are highly perishable products, and their processing and preservation technologies that ensure longevity and freshness are of great importance [37]. Drying and preservation of mushrooms is important to prevent enzymatic and non-enzymatic browning, minimize moisture-mediated reactions, and achieve their unique flavor, texture and nutritional value [37]. Some of these methods are carried out under harsh conditions [34]. Common drying methods include solar, vacuum, freezing, hot-air, microwave, infrared and ultrasound-assisted drying [30,32,37]. It has been reported that the ergosterol content in dried mushrooms is significantly lower than in fresh mushrooms [34]. In addition, there is a significant difference in ergosterol content between air-dried and freeze-dried mushrooms [30] (Table 2). The decrease in ergosterol content during storage occurs continuously, with or without exposure to light, suggesting that conversion to vitamin D is not the only cause of the decrease in content. Furthermore, this study indicates enzymatic degradation of the ergosterol side chain at room temperature, rather than isomerization by UV radiation [34]. This enzymatic activity is absent when fungi are treated at high temperatures above 90 °C after the enzyme has been denatured [34]. Guan et al. [32] additionally reported oxidative degradation of ergosterol during the period.

In addition, differences in extraction methods, temperature, solid-to-solvent ratio and number of extractions, as well as the pH of extraction solutions, can lead to significant differences in the ergosterol content in the same samples [9,29,38].

Button mushrooms are one of the most widely cultivated mushrooms in the world, especially in Serbia, and one of the objectives of this study was to estimate the ergosterol content in different parts (caps and stems) of this type of mushroom. The results are presented in Table 3. The distribution of ergosterol content in the different parts varies between 0.43 and 1.61 mg/g and between 1.66 and 8.44 mg/g for caps and stems, respectively. In general, the stems contain one to nine times higher amounts of ergosterol than the caps. According to the literature, the caps, especially the gills, contain significantly higher [13] or similar concentrations of ergosterol to the stems [32]. Interestingly, this ratio changes in favor of the stems during storage. In a study reported by Guan in 2016 [32], the ergosterol concentration was significantly higher in the stems than in the caps after 14 days of storage of fresh mushrooms.

Ergosterol is a sterol that plays an important role in various cellular metabolic and physiological functions, including the growth and reproduction of fungi [4,6,7,20]. The ergosterol biosynthetic pathway involves several metabolic steps involving the activity of 20 different enzymes and metalloenzymes, with Fe and Zn being important cofactors [20]. In general, the correlation between metal concentrations in fungi is influenced by various factors, including the composition of the substrate (compost or soil), environmental and growth conditions, competition with other elements through specific uptake mechanisms, and the fungal species [39]. It should be emphasized that the ability of fungi to selectively absorb inorganic elements is generally characterized by the uptake of elements considered physiologically necessary for the species, such as Zn, Cu and Fe, by the mycelia and their deposition in the bodies [15].

To find a possible correlation between ergosterol and metal concentrations, Cu, Fe and Zn were measured in all estimated fungal samples. The results are shown in Table 1.

The highest Fe content was found in *Agaricus bisporus* from Serbian markets. The iron values determined were in agreement with the reported Fe values of *Agaricus bisporus* from Turkey [40]. With a mean value of 75.4 mg/kg DM, the wild-growing species had a lower Fe content than the marketed mushrooms. The bioavailability of iron in mushrooms is very high, so that more than 90% of the iron present can be absorbed by the human body [41].

The zinc content varies greatly between 25 and 200 mg/kg DM, and even higher levels have been found in some species [21]. Median values of 98.6 and 104 mg/kg DM were determined for 87 ectomycorrhizal and 43 saprobic species, respectively [42]. According to the literature, the Zn concentration in fungi is higher than that of Cu [43]. The Cu and Zn concentrations reported here are within the range observed in wild species in other parts of the world, such as Poland (Cu: 36–59, Zn: 74–110 mg/kg DM) [44] and Turkey (Cu: 19–65, Zn: 45–198 mg/kg DM) [45]. The highest Cu content was found in the wild species *B. appendiculatus* (93.03 mg/kg DM). The background copper content in most European species of wild edible mushrooms from uncontaminated areas is between 20 and 100 mg/kg DM [21]. The average Cu content was higher in wild mushrooms than in cultivated species from Korean markets (28 and 12 mg/kg DM, respectively) (Table 1), which is consistent with previous results [46]. Zinc levels were in the range of 14–150 mg/kg DM, 13–77 mg/kg dm and 50–140 mg/kg DM in wild, Korean and Serbian mushrooms, respectively. The zinc values determined were consistent with the reported Zn values in uncontaminated regions [15,41,46].

The results of the Pearson correlation analysis showed a significant positive correlation (*p* ˂ 0.01) between ergosterol and Fe concentration. Copper and ergosterol are negatively correlated at the *p* ˂ 0.05 level, and a significant positive correlation (*p* ˂ 0.01) was found between Cu and Zn. There was no significant correlation between ergosterol and Zn content. In order to highlight the possible relationship between the investigated parameters, the data obtained were additionally subjected to a principal component analysis (PCA). The PCA analysis identified two factors that explained 79.14% of the total variance. The first principal component explained 45.12% of the total variance in ergosterol and Fe, which probably indicates a direct influence of Fe concentration on ergosterol biosynthesis. Zinc and Cu were in the second component (34.01%), probably indicating their correlation in the growth substrate and a similar absorption mechanism by fungi [47]. The Kaiser–Meyer–Olkin (KMO) measure of sampling adequacy for this dataset was 0.5. The Bartlett’s test for sphericity was also reliable, with a chi-square of 17.75.

Iron is a component of cytochrome P450 enzymes and squalene epoxidase, and zinc is involved in the final stages of ergosterol synthesis as a component of sterol reductase [20,48]. In addition, excess Zn and Cu could have a negative effect on ergosterol biosynthesis by inducing oxidative stress or inhibiting the enzymes of the mevalonate pathway [20]. This suppression of ergosterol production is a key role in the action of azole antifungals. In addition, some copper salts are widely used in agriculture and medicine to inhibit fungal growth by suppressing ergosterol synthesis [49,50]. It has also been reported [18] that heavy metals, including Cu and Zn, can negatively affect the production of antioxidant enzymes and some thiol components in *A. bisphorus*, disrupting metabolic processes, possibly including the ergosterol synthesis pathway. Regarding future perspectives, the correlations between ergosterol content and elemental concentration obtained in this study are certainly just part of a preliminary phase and could be the subject of an independent and focused study.

## 3. Materials and Methods

### 3.1. Samples

In order to compare the ergosterol contents in mushrooms, three different mushroom sources were selected: cultivated mushrooms from two grocery stores and wild mushrooms. Two different markets were selected: Korean and Serbian markets. Fresh shiitake (*Lentinula edodes*), oyster (*Pleurotus ostreatus*), button (*Agaricus bisporus*), shimeji (*Hypsizygus marmoreus*), Jew’s ear (*Auricularia auricula-judae*), king oyster (*Pleurotus eryngii*), giant oyster (*Pleurotus giganteus*) and enoki mushrooms (*Flammulina velutipes*) were purchased from Korean grocery stores. Only Agaricus bisporus mushrooms were purchased in Serbian stores. Wild edible mushrooms from the Boletus family (*Boletus appendiculatus*, *Boletus edulis*, *Butyriboletus fechtneri*, *Phylloporus rhodoxanthus*, *Leccinum crocipodium* and *Boletus impolitus*) are very popular in Serbia. All these mushrooms were purchased from mushroom pickers.

The mushrooms were cut into pieces, mixed and pooled for the determination of metal and sterol contents. For ergosterol determination in different parts of the mushrooms, the button mushrooms were cleaned with a plastic knife without washing and separated into caps and stems.

Given the aim to convert the measured concentrations to dry weights, the water content of the mushroom samples was determined by drying them in an oven at 105 °C until a constant weight was reached. The water contents of the analyzed samples were in agreement with a consensus that the mean water content of mushrooms is about 90% [51].

### 3.2. Chemicals and Reagents

Ergosterol, dimethyformamide (DMF), acetonitrile (ACN), dimethyl sulfoxide (DMSO), dichloromethane (DCM), methanol (MeOH) and tetrabutylammonium hexafluorophosphate (TBAHFP) were purchased from Sigma Aldrich (St. Louis, MO, USA). Nitric acid (65% p.a.), hydrogen peroxide (30% p.a.) and all other chemicals were of analytical grade and supplied by Merck (Darmstadt, Germany). A multi-element stock solution containing 1.000 g/L of the elements (Fe, Cu and Zn) was used to prepare standard solutions for ICP-OES measurements. Standard stock solutions were used to prepare calibration standard solutions after appropriate dilution with 1.0% *v*/*v* nitric acid and ultra-pure reverse osmosis water with a resistance of 18.2 MΩ/cm from a MilliQ plus system (Millipore, Bedford, USA). Certified reference material ERM - CD281 [52] was used to verify the accuracy and precision of the ICP-OES instruments.

### 3.3. Ergosterol Extraction

To determine the ergosterol contents, the mushroom samples were pulverized. The extraction was carried out with a solvent mixture of methanol–dichloromethane (75:25 *v*/*v*), and the ratio of the solid to the liquid phase was 1:25 (*w*/*w*). The samples were shaken at room temperature for 30 min, and then the extracts were centrifuged at 10,000 rpm for 5 min. The extraction procedure was repeated three times. The total extracts were evaporated to dryness, and the extract residues were dissolved in 10 mL acetonitrile.

### 3.4. Voltametric Analysis of Ergosterol

The voltametric measurements were carried out with a potentiostat/galvanostat CHI 760b (CH Instruments, Austin, TX, USA). A boron-doped diamond (BDD) electrode (Windsor Scientific Ltd., Slough, UK) was used as the working electrode, and an Ag/AgCl (non-aqueous, inner solution: 0.01 AgNO_3_-containing carrier electrolyte) was used as the reference electrode and platinum wire was used as counter electrode. The working cell with a total volume of 5 mL was equipped with a standard three-electrode system.

The working electrode proposed by the supplier had a resistance of 0.075 Ω cm and a boron doping of 1000 ppm. Before each working day, the electrode was treated with 1 M sulfuric acid at a potential of +2 V for 180 s and then again at a potential of −2 V for 180 s. Before each measurement, the electrode was lightly cleaned with a piece of cotton. A pH meter (model: Orion 1230) with a combined glass electrode (model: Orion 9165BNWP, Thermo Fisher Scientific, Waltham, MA, USA)) was used to measure all pH values. All experiments were carried out at room temperature, i.e., at 20 °C. Each sample was analyzed in duplicate, and each analysis consisted of three replicates. LOD and LOQ values were determined as three and ten times the standard deviation of the blank solutions, respectively.

### 3.5. Sample Preparation for Elemental Analysis

To determine the metal contents, the mushrooms were prepared by microwave-assisted digestion using a Berghof microwave oven (Speedwave, Berghof, Germany). The microwave digestion system was equipped with 12 PTFE vessels. The procedure was as follows: 0.50 g of dried mushrooms was weighed into the microwave vessel, then 5 mL of 65% HNO3 and 3 mL of 30% H_2_O_2_ were added. The digestion was carried out according to the following program: heating to 190 °C for 10 min and holding at this temperature for 15 min. After cooling, the samples were quantitatively transferred to a volumetric flask and diluted to 50.0 mL with ultra-pure water.

### 3.6. Instrumental and Operating Parameters for ICP-OES Analysis

The measurements of iron, copper and zinc were carried out in an inductively coupled plasma atomic emission spectrometer (ICP-OES) (model: 6500 Duo (Thermo Scientific, Altrincham, UK)) equipped with a CID86 chip detector. This instrument operates sequentially with radial and axial torch views. The flow rates for the auxiliary argon and concentric nebulizer were 0.5 L/min. The sample flow rate was 1.0 mL/min. The entire system was controlled by Iteva software. The selected wavelengths (nm) for the elements were as follows: Fe (259.9), Cu (324.7) and Zn (213.8). Each sample was analyzed in duplicate, and each analysis consisted of three replicates. LOD and LOQ values were determined as three and ten times the standard deviation of the blank solutions, respectively.

### 3.7. Statistical Analysis

Descriptive statistics, Pearson correlation analysis and principal component analysis (PCA) were performed with statistical software SPSS 11.0 (SPSS Inc., Chicago, IL) and used to estimate the linear dependencies between the variables and to identify latent factors among them.

## 4. Conclusions

The results obtained in the present study show that the ergosterol contents of the mushrooms investigated vary. Wild-growing mushrooms generally contain less ergosterol than cultivated mushrooms. In cultivated mushrooms, the ergosterol content strongly depends on the mushroom species, cultivation conditions and storage time, followed by the country of origin. The results on the distribution of the ergosterol content between the different parts (stems and cups) of button mushrooms show that the ergosterol content was significantly higher in the stems.

The results of the Pearson correlation analysis and principal component analysis (PCA) with two extracted PCs, which explained 79.14% of the total variance, indicate a significant relationship between ergosterol and Fe, Zn and Cu contents. The first principal component indicates with high probability a direct influence of Fe on ergosterol biosynthesis. In addition, the extraction of Zn and Cu in PC2 indicates their correlation in the grown substrate and a similar absorption mechanism of the fungus, as well as the possibility of a negative effect of Cu on ergosterol content.

## Figures and Tables

**Table 1:** Ergosterol (mg/g DM) and microelement concentrations (mg/kg) in different types of wild and cultivated edible mushrooms.

Origin	Family Name	Botanical Name	Common Name	Ergosterol	Fe	Zn	Cu
Wild-grown, Serbia	*Boletaceae*	*Phylloporus rhodoxanthus*	Gilled bolete	0.13	10.55	43.51	5.46
		*Boletus edulis*	Penny bun	0.71	45.83	17.64	7.91
		*Boletus appendiculatus*	Butter bolete	0.01	44.98	49.92	5.74
		*Boletus appendiculatus*	Butter bolete	0.13	55.99	103.4	93.03
		*Phylloporus rhodoxanthus*	Gilled bolete	˂LOD	102.1	51.31	33.06
		*Leccinum crocipodium*	Saffron bolete	1.06	49.8	66.10	63.66
		*Butyriboletus fechtneri*	Pale bolete	7.04	393	68.22	25.71
		*Boletus impolitus*	Iodine bolete	0.35	26.76	149.7	5.02
		*Boletus impolitus*	Iodine bolete	˂LOD	16.54	13.71	2.91
		*Boletus edulis*	Penny bun	0.61	8.50	27.12	33.70
Cultivated, Serbia	*Agaricaceae*	*Agaricus bisporus*	White button	1.43	210.3	52.32	26.03
		*Agaricus bisporus*	White button	1.14	265.1	84.21	38.28
		*Agaricus bisporus*	White button	1.69	3204	50.32	19.41
		*Agaricus bisporus*	White button	3.92	325.2	140.2	65.19
		*Agaricus bisporus*	White button	4.67	216.4	65.84	25.04
Cultivated, Korea	*Physalacriaceae*	*Flammulina velutiper*	Enoki	0.54	47.48	41.92	5.12
	*Lyophyllaceae*	*Hypsizygus marmoreus*	Shimeji	1.46	44.33	44.12	6.90
	*Agaricaceae*	*Agaricus bisporus*	White button	1.90	24.35	37.63	4.44
	*Pleurotaceae*	*Pleurotus giganteus*	Giant oyster	1.83	61.34	77.31	21.74
	*Lyophyllaceae*	*Hypsizygus marmoreus*	Shimeji	2.43	54.65	67.50	42.27
	*Omphalotaceae*	*Lentinula edodes*	Shitake	5.90	46.70	51.71	6.07
	*Agaricaceae*	*Agaricus bisporus*	White button	6.96	479.9	55.14	23.81
	*Auriculariaceae*	*Auricularia auricula-judae*	Wood ear, Jew’s ear	1.99	47.32	13.13	1.62
	*Pleurotaceae*	*Pleurotus ostreatus*	Oyster	4.48	10.24	60.91	5.67
	*Pleurotaceae*	*Pleurotus eryngii*	King oyster	3.16	76.61	32.52	5.29
Minimum				0.01	8.50	13.12	1.62
Maximum				7.04	479.9	149.7	93.03
Mean				2.39	119.3	58.59	22.88
Standard deviation				2.16	135.6	33.64	23.29

LOD: Limit of detection.

**Table 2:** Average ergosterol concentrations in different types of edible mushrooms from different regions around the world.

Botanical Name	Common Name	Storage Condition	Ergosterol	Country	Reference
*Lentinula edodes*	Shitake	Fresh	1179.74 µg/g	China	[28]
*Lentinula edodes*	Shitake	Fresh	6820 µg/g	India	[26]
*Agaricus bisporus*	White button	Fresh	1.1–36.1 mg/100 g	Poland	[31]
	Brown button	Fresh	26.68 mg/100 g		
*Lentinula edodes*	Shitake	Fresh	4.59 mg/g	China	[27]
*Auricularia auricula-judae*	Jew’s ear	Fresh	4.22 mg/g		
*Agaricus bisporus*	White button (caps)	Fresh	6.12 mg/g	Canada	[32]
	White button (stems)		5.20 mg/g		
	Brown button (caps)	Fresh	7.59 mg/g		
	Brown button (stems)		7.56 mg/g		
*Agaricus bisporus*	Button	Fresh	9.85 mg/g	Poland	[30]
*Pleurotus ostreatus*	Oyster	Fresh	7.64 mg/g		
*Lentinula edodes*	Shitake	Fresh	9.33 mg/g		
*Agaricus bisporus*	Button	Air-dried	8.61 mg/g		
*Pleurotus ostreatus*	Oyster	Air-dried	7.54 mg/g		
*Lentinula edodes*	Shitake	Air-dried	8.59 mg/g		
*Agaricus bisporus*	Button	Freeze-dried	9.88 mg/g		
*Pleurotus ostreatus*	Oyster	Freeze-dried	8.79 mg/g		
*Lentinula edodes*	Shitake	Freeze-dried	8.94 mg/g		
*Agaricus bisporus*	Button		6.9 mg/g	Italy	[29]
*Agaricus bisporus*	Portobello	Fresh	7.3 mg/g		
*Pleurotus ostreatus*	Oyster		4.9 mg/g		
*Agaricus bisporus*	White button	Fresh	2429.68–6995.30 µg/g	India	[9]
*Agaricus bisporus*	White button	Fresh	3.8 mg/g	Spain	[33]
*Pleurotus ostreatus*	Oyster	Fresh	774.2–762.4 mg/100 g	Korea	[21]
*Lentinula edodes*	Shitake		515.8 mg/100 g		

**Table 3:** Ergosterol concentrations (mg/g DM) in different parts of Serbian button mushrooms.

	Button Mushroom (Stem)	Button Mushroom (Cap)
Ergosterol (mg/g)	3.058	0.634
	3.981	0.434
	1.655	0.729
	1.662	1.057
	2.19	1.603
	2.041	1.338
	8.441	1.451
	4.255	1.131
	2.883	1.614
	1.717	0.705
	7.001	1.149
Minimum	1.655	0.434
Maximum	8.441	1.614
Mean	3.53	1.08
Standard deviation	0.686	0.122

## Data Availability

The dataset is available on request from the authors.
